# Validation of the Short Physical Performance Battery via Plantar Pressure Analysis Using Commercial Smart Insoles

**DOI:** 10.3390/s23249757

**Published:** 2023-12-11

**Authors:** Chan Woong Jang, Kyoungmin Park, Min-Chul Paek, Sanghyun Jee, Jung Hyun Park

**Affiliations:** 1 Department of Rehabilitation Medicine, Gangnam Severance Hospital, Rehabilitation Institute of Neuromuscular Disease, Yonsei University College of Medicine, Seoul 06229, Republic of Korea; dearjcw89@gmail.com (C.W.J.); min768@yuhs.ac (M.-C.P.); shjee1015@yuhs.ac (S.J.); 2 Department of Medical Device Engineering and Management, The Graduate School, Yonsei University College of Medicine, Seoul 06229, Republic of Korea; km345754@yuhs.ac; 3 Department of Integrative Medicine, The Graduate School, Yonsei University College of Medicine, Seoul 06229, Republic of Korea

**Keywords:** aged, foot orthoses, frailty, physical functional performance

## Abstract

This cross-sectional study, conducted at a tertiary care hospital’s rehabilitation clinic, aimed to validate Short Physical Performance Battery (SPPB) results obtained through plantar pressure analysis using commercial smart insoles (SPPB-SI) and to compare these results to manually acquired results by an experienced examiner (SPPB-M). This study included 40 independent-walking inpatients and outpatients aged 50 or older. SPPB-SI and SPPB-M were administered concurrently, with the smart insoles providing plantar pressure data that were converted into time–pressure curves. Two interpreters assessed the curves, determining component completion times for the SPPB-SI scores. Among the 40 participants (mean age: 72.98, SD: 9.27), the mean total SPPB-SI score was 7.72 ± 2.50, and the mean total SPPB-M score was 7.95 ± 2.63. The time recordings and measured scores of each SPPB-SI component exhibited high reliability with inter- and intra-interpreter correlation coefficients of 0.9 and 0.8 or higher, respectively. The intraclass correlation coefficient between the total SPPB-SI and SPPB-M scores was 0.831 (*p* < 0.001), and that between the component scores of the two measurements ranged from 0.837 to 0.901 (*p* < 0.001). Consistent correlations with geriatric functional parameters were observed for both SPPB-SI and SPPB-M. This study underscores the potential of commercial smart insoles as reliable tools for conducting SPPB assessments.

## 1. Introduction

The Short Physical Performance Battery (SPPB), first described in 1994 by Guralnik et al., is a clinical examination tool used to measure the functional status and physical performance of the lower extremities [1]. It consists of three components: the balance, gait speed, and five chair stand tests [1]. The score ranges from 0 (worst) to 12 (best), with higher scores indicating better function [1]. The SPPB has primarily been utilized in the evaluation of older patients and has been identified as a diagnostic criterion for geriatric syndromes in previous studies [2,3,4].

In the standardized protocol of the SPPB, each component is measured using a manual method that involves employing a stopwatch [5,6]. The score is calculated by an examiner who manually records the time taken for the completion of each test using a handheld stopwatch, based on a predefined criterion [1]. However, this measurement method is susceptible to human error, potentially causing variations in the results owing to the examiner’s skills [7]. Currently, to overcome the limitations of the manual approach, there are ongoing endeavors to develop novel devices, including sensors that can offer more objective measurements for the SPPB and its components [7,8,9,10,11].

Nevertheless, these new devices have their own drawbacks. For instance, items such as belts, glasses, pendants, etc., come with the inconvenience of needing to be reapplied for each measurement [9,10,11]. There is also a vulnerability in that the fixed position of devices during measurement can change, and users unfamiliar with such devices may find it necessary to make a new purchase. Furthermore, other devices have the drawback of requiring additional space for use [7,8]. In comparison to the conventional stopwatch method, these factors highlight clear limitations in terms of commercialization and the feasibility of conducting continuous examinations in older community-dwelling adults.

Smart insoles capable of capturing real-time measurements of plantar pressure during everyday activities have been introduced and made available in the market at reasonable prices [12,13]. Since the inception of smart insoles, numerous studies have explored their applications in the medical field. These studies encompass a wide range of areas, including but not limited to gait and posture stability analysis, the prevention of foot wounds in individuals with diabetes, and the assessment of fall risk in older adults by analyzing the distribution of plantar pressure [14,15,16,17,18,19]. Notably, several studies have reported high accuracies for gait and postural analyses using real-time plantar pressure monitoring, leading to a gradual expansion of applications for smart insoles [20].

Considering the benefits of smart insoles, such as high accuracy, low cost, low power consumption, and mobility, we hypothesized that they could be suitable alternatives to the current standardized protocol for SPPB testing. To confirm this, we aimed to validate the SPPB results by measuring plantar pressure changes using commercial smart insoles and comparing them with those obtained by an examiner using a stopwatch. Based on these results, we propose the integration of smart insoles as a novel system for measuring the SPPB.

## 2. Materials and Methods

### 2.1. Study Population

This study prospectively enrolled participants from both inpatient and outpatient rehabilitation clinics at the Gangnam Severance Hospital between January and May 2023. Eligible participants were individuals aged 50 years or older who were capable of walking independently, with or without a gait aid, and possessed sufficient cognitive function to perform the required tests as per medical instructions. Individuals who were unable to walk, had acute medical or surgical conditions, or had a terminal illness with a life expectancy of less than 6 months were excluded. Patients with cognitive impairments that prevented them from completing consent forms or following medical staff instructions were also excluded. The study protocol was approved by the Institutional Review Board of Gangnam Severance Hospital (IRB No. 3-2022-0440), and all participants provided written informed consent.

### 2.2. Commercial Smart Insoles

In this study, we utilized commercially available smart insoles I-SOL^®^, manufactured by Gilon (Seongnam, Republic of Korea), to collect plantar pressure data. Previous studies have demonstrated that these devices accurately measure changes in plantar pressure during walking [21]. Additionally, it has been revealed that they can be utilized for real-time walking speed estimation, weight estimation, and activity classification [12]. For this study, we obtained a program from the manufacturer to receive real-time plantar pressure data.

**Figure A1:**
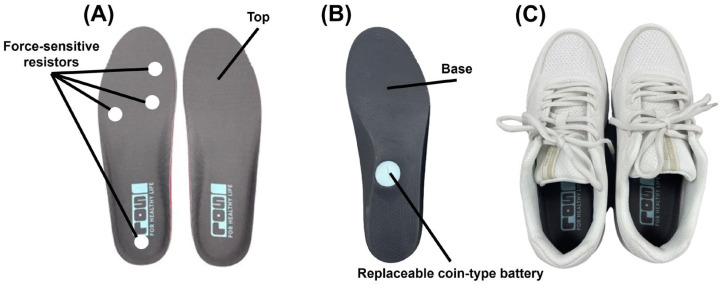
(**A**) The top of the smart insoles, I-SOL^®^ (Gilon, Seongnam, Republic of Korea), (**B**) the base, and (**C**) the layout of the inserted smart insoles.

As illustrated in Figure A1, each insole was equipped with four circular force-sensitive resistor (FSR) sensors, with a diameter of 14 mm each, and a triaxial accelerometer sensor. Additionally, the insoles contained a low-energy Bluetooth module and replaceable coin-type battery. The FSR sensors precisely detected changes in force at four specific key points, and the accelerometer sensors accurately captured acceleration along the X-, Y-, and Z-axes. The data were collected at a frequency of 40 Hz using a Raspberry Pi 3 system.

The smart insole had a thickness of 6.0 mm at the front, 10.5 mm in the middle, and 10.0 mm at the back, with a total weight of 60 g. It was offered in a range of sizes, increasing in 5 mm increments, from 230 mm to 280 mm. Each participant had the opportunity to choose the size that best suited their feet. 

### 2.3. SPPB Measurement (the Balance, Gait Speed, and Five Chair Stand Tests)

This study involved simultaneous administration of the SPPB measurement using smart insoles (SPPB-SI) and the manual SPPB measurement using a stopwatch (SPPB-M). Prior to the commencement of this study, the examiner, with over five years of experience conducting the SPPB following standardized protocols, underwent specific training to administer the SPPB by measuring the performance of five participants. The measurements consisted of three tests: the balance, gait speed, and five chair stand tests. For the SPPB-SI, the participants performed each component of the SPPB while wearing the smart insoles. Concurrently, for the SPPB-M, an examiner administered the SPPB using a standardized protocol, relying on a stopwatch as described in a previous study [1]. Before starting the test, the participants rolled several steps in place to assess their comfort with the smart insoles and to confirm that the change in plantar pressure was normally recognized.

The balance test assessed participants’ standing balance in three positions: side-by-side, semi-tandem, and tandem stances. The participants were instructed to maintain each position for up to 10 s, utilizing their trunk and arms for stability if necessary. The semi-tandem stance was performed first; if it could not be sustained for more than 10 s, the side-by-side position was tested. The tandem stance was assessed if the semi-tandem stance was successfully maintained for more than 10 s. The duration of each position was recorded.

The gait speed test measured participants’ walking speed over a 4-m distance, with or without the use of a gait aid. Participants were instructed to initiate their walk precisely from the beginning of the 4-m mark and to conclude precisely at the end of the 4-m mark, with no buffer space before or after the marked distance. The examiner recorded the time taken to complete the 4-m distance or the maximum time if the distance was not fully covered.

During the five chair stand test, participants were instructed to stand up and sit down five times as quickly as possible with their arms crossed. The time required to complete the task was recorded.

Furthermore, the scores of each component of the SPPB were calculated based on established cutoff points, as described in a prior study [1]. A summary performance scale was generated by summing the scores from the three component tests to provide an overall assessment.

### 2.4. Interpretation of Plantar Pressure Graphs

The plantar pressure obtained from the FSR sensor was converted to kilopascal (kPa) units using the following equation: Pressure kPa = 10.7835 + 0.0587 × FSR + 0.0004 × FSR^2^. Then, we aggregated the pressure data collected from the four FSRs on each left and right foot separately. The time-dependent changes in the combined plantar pressure data were illustrated as time–pressure graphs for each foot.

Two independent interpreters, who were blinded to the demographic and clinical parameters of the participants, reviewed the time–pressure curve graphs for each side and determined the start and end points of the test based on changes in the graph pattern. In a pilot test involving five individuals, we confirmed a delay of approximately 0.3 s in data collection when using smart insoles. Nonetheless, this delay did not pose any issues with result interpretation as the graphical analysis focused on verifying the start and end points. In cases where establishing these points was challenging, the interpreters engaged in discussions and reached a mutual agreement. For the interpretation, 200 ms vertical grids were introduced in the graphs to determine the time points.

After determining the time elapsed between the measured start and end points, we calculated the SPPB-SI based on the cutoff times for the three components of the SPPB [1].

### 2.5. Covariables

Various functional geriatric parameters were estimated to assess the clinical relevance of the SPPB-SI results. For frailty status, we used the Korean version of the Fatigue, Resistance, Ambulation, Illnesses, and Loss of Weight (K-FRAIL) scale. Individuals who selected 1–2 positive items were considered prefrail and those who selected 3–5 positive items were considered frail [22,23]. It was demonstrated to be non-inferior to the existing standard of the 28-item Kihon + 3 index and exhibited greater sensitivity and lower specificity compared to the original Kihon checklist [22]. Grip strength was measured using a handgrip dynamometer, with participants seated and their elbows resting on a table. The maximum grip strength of both arms, measured twice for each arm, was used for the analysis. Functional capacities and activities of daily living were determined using the Korean version of the modified Barthel index (K-MBI) which is a translation based on the 5th version of the MBI [24,25]. Fall risk was assessed using the Korean version of the Berg Balance Scale (K-BBS), which is a reliable assessment tool [26]. This involved translating the original BBS into Korean and subsequently retranslating it into English for evaluation, confirming its high reliability [27]. Additionally, functional ambulatory category (FAC) was used to determine the amount of physical support required for walking [28]. Cognitive function was assessed using the Korean Mini-Mental State Examination (K-MMSE), which has been shown to be clinically applicable for elderly patients based on established normal values [29,30].

### 2.6. Statistical Analysis

The sample size was calculated using tables based on Zou’s formulas to evaluate the intraclass correlation coefficient (ICC) between the SPPB-SI and SPPB-M [31]. With an ICC of 0.80 assumed as a priori, a study power of 0.80 (1-β), and a 95% confidence interval half-width of less than 0.15, this study required 35 participants. To account for a possible 10% dropout rate or exclusion during the testing process, our goal was to recruit 40 participants.

We employed the Pearson correlation coefficient and κ coefficient to establish the reliability between the time and scores recorded by the two interpreters for the SPPB based on the plantar pressure graph, respectively. To visualize the concordance between the time measurements, we created a Bland–Altman plot, which illustrates the difference plotted against the average. Subsequently, we evaluated the ICCs and κ coefficients of the total and component scores between the SPPB-SI and SPPB-M. ICC estimates and their 95% confident intervals were calculated based on a single rater, absolute-agreement, and two-way mixed-effects model. Scatterplots with jitters and linear regressions were used to examine correlations between the total and component scores of both SPPB measurements. Furthermore, we utilized the Spearman’s correlation coefficient to evaluate the associations between the SPPB-SI parameters and geriatric functional parameters. The R statistical package version 4.1.2 (R Foundation for Statistical Computing, Vienna, Austria) was used to perform all data analyses. Statistical significance was set at *p* < 0.05.

## 3. Results

A total of 40 individuals (24 women and 16 men) were included in this study. The basic characteristics of the participants are summarized in Table 1. All participants were able to walk independently, although four required a walking aid. Despite this, they were relatively fit, as indicated by a mean K-FRAIL score of 1.55 (1.13), a mean K-BBS score of 43.55 (12.61), and a mean FAC score of 4.03 (1.23). Of the total participants, 16 were able to perform their daily activities independently, with a K-MBI score of 100 points. The average K-MBI score was 86.83 (16.74) points. Furthermore, participants scored an average of 27.15 (1.78) points on the K-MMSE, indicating no limitations in their ability to direct and perform the test. There were no adverse events, including falls or any pain associated with participating in the tests.

**Figure 1:**
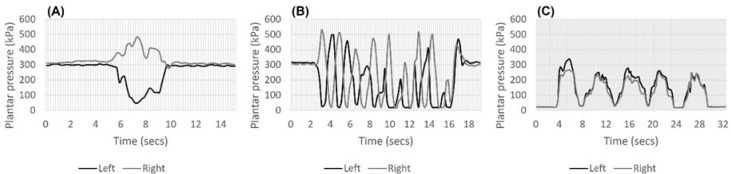
Examples of the plantar pressure graphs for the Short Physical Performance Battery. (**A**) The balance, (**B**) gait speed, and (**C**) five chair stand tests.

**Figure A2:**
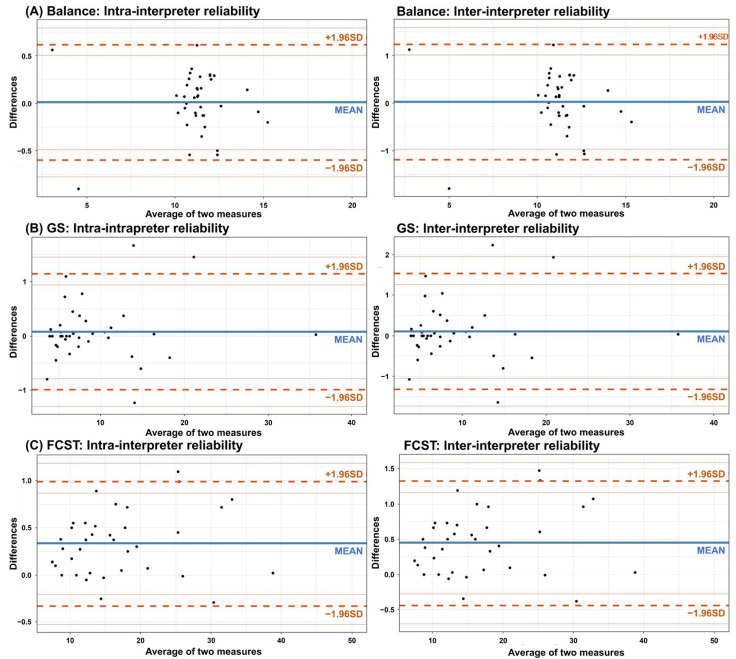
Bland–Altman plots for intra- and inter-interpreter reliability of the Short Physical Performance Battery times measured through plantar pressure graph interpretation using smart insoles. The solid horizontal line represents the mean difference and the dotted lines represent the 95% limits of agreement between the two measurements. (**A**) The balance (Balance), (**B**) gait speed (GS), and (**C**) five chair stand (FCST) tests.

Examples of the plantar pressure graphs for each SPPB component are shown in Figure 1. The results of the intra- and inter-interpreter reliability of SPPB-SI time and scores are presented in Table 2. The time recordings of each SPPB-SI component, derived from the interpretation of the plantar pressure graph, exhibited outstanding reliability with Pearson correlation coefficients of 0.9 or higher. A Bland–Altman concordance analysis illustrated relatively small dispersion for both the intra- and inter-interpreter reliability of the time recordings (Figure A2). All the mean differences between the two measurements demonstrated very good agreement, with the mean difference staying within 0.5. The component of SPPB-SI scores based on time recordings demonstrated excellent reliability, with inter- and intra-interpreter κ coefficients of 0.8 or higher.

**Figure 2:**
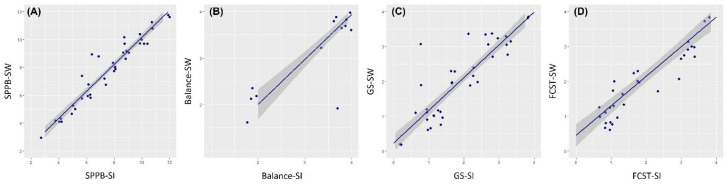
Scatterplots with jitters and linear fitted lines, including 95% confidence intervals, for the Short Physical Performance Battery (SPPB) scores manually measured using a stopwatch (M) and smart insoles (SI). (**A**) The total SPPB, (**B**) balance (Balance), (**C**) gait speed (GS), and (**D**) five chair stand (FCST) scores.

The mean total SPPB-SI and SPPB-M scores of the participants were 7.72 (2.50) and 7.95 (2.63), respectively. The ICC between the average total SPPB-SI and SPPB-M scores was 0.831 (*p* < 0.001). The mean balance test scores for SPPB-SI and SPPB-M were 3.76 (0.63) and 3.68 (0.73), respectively, with an ICC of 0.896 (*p* < 0.001) between the two measurements. The mean gait speed test scores for SPPB-SI and SPPB-M were 2.08 (1.19) and 2.18 (1.17), respectively, with an ICC of 0.901 (*p* < 0.001) between the two measurements. The mean five chair stand test scores for SPPB-SI and SPPB-M were 2.13 (1.34) and 2.25 (1.26), respectively, with an ICC of 0.837 (*p* < 0.001) between the two measurements. Correlations between the SPPB-SI and SPPB-M parameters, using scatterplots with random jitters on the data points and a linear fitted line, are displayed in Figure 2. The shaded areas represent the 95% confidence intervals. Also, the κ coefficients were 0.940 for the balance test scores, 0.881 for the gait speed test scores, 0.976 for the five chair stand test scores, and 0.934 for the total scores.

When considering an SPPB score of 9 or less as a vulnerable state, 28 and 27 individuals were classified as vulnerable by the SPPB-SI and SPPB-M measurements, respectively. The κ value for classifying vulnerability between the two measurements was 0.886 (*p* < 0.001).

To evaluate the content validity of the SPPB-SI and SPPB-M scores, we analyzed the correlations between the SPPB parameters and commonly employed functional parameters in geriatric assessments. These measurements included the K-FRAIL, grip strength, K-MBI, K-BBS, FAC, and K-MMSE scores. Correlations with these functional parameters remained consistent for both the SPPB-SI and SPPB-M scores (Table 3).

## 4. Discussion

This study demonstrated a robust agreement between the total and component scores of SPPB-SI and SPPB-M, as well as their counterparts, among the study participants. Furthermore, both the SPPB-SI and SPPB-M scores aligned with the geriatric functional parameters. Additionally, we observed a high level of agreement between the measured test times obtained from a plantar pressure graph using smart insoles and the manual method. The significance of our study lies in being the first to validate the accuracy and propose the applicability of SPPB using smart insoles.

Several studies have previously explored the use of plantar pressure distribution obtained from smart insoles as a marker [20]. Specifically, a study performed in older patients demonstrated that smart insoles can be utilized to predict fall risks and frailty [32,33,34]. More recent studies have expanded the application of plantar pressure analysis using smart insoles to include disease diagnosis. For instance, one study demonstrated the potential for the early detection of Parkinson’s disease using smart insoles, whereas another study suggested the use of smart insoles to predict and classify sarcopenia [17,35]. Ongoing efforts also aim to utilize smart insoles for treatment purposes, involving monitoring plantar pressure and inducing alterations in its distribution [36].

Although smart insoles have gained traction in the medical field, there has been a notable absence of analysis regarding their utilization for conducting the SPPB. As society ages, evaluating the functional abilities of older individuals becomes increasingly important. In this context, the SPPB is widely used and has proven to be an effective assessment tool [37]. In other words, the ability to measure the SPPB more conveniently and accurately in everyday life carries significant implications for an aging society. Thus, our study’s successful demonstration of conducting the SPPB through plantar pressure analysis using smart insoles not only introduces the feasibility of this approach but also establishes a solid foundation for the effortless integration of the SPPB into diverse clinical settings and research initiatives.

Our findings provide compelling evidence to support these claims. The SPPB results, whether obtained through manual measurement using a stopwatch or via smart insoles, consistently demonstrated a significant level of agreement. Scores for each component of the SPPB also showed a comparable level of consistency. Conducting both tests simultaneously, with smart insoles inserted into the patient’s shoes during the examination, minimizes the likelihood of influencing the results. Therefore, the substantial agreement between the two tests suggests that smart insoles can effectively replace the traditional stopwatch method.

Furthermore, maintaining a consistent correlation with various geriatric functional assessment parameters adds significance to our study results. Additionally, a high level of agreement was observed in the evaluation identifying vulnerable status based on the total SPPB score. This not only confirms measurement accuracy but also indicates that smart insoles can serve as a viable alternative for assessing the clinical status of patients. The potential for such substitution is noteworthy, establishing a foundation for continuous assessment of the function of the lower extremities of elderly patients during their daily lives using smart insoles.

In this study, two independent interpreters were responsible for manually analyzing the plantar pressure graphs obtained from the smart insoles. Although the results indicated a notably high level of reliability both within and between interpreters concerning the timing and scoring of the SPPB, it is important to acknowledge that the analysis of plantar pressure graphs presents a significant challenge for future research. We believe that employing an automated algorithm, utilizing data from the plantar pressure fluctuation graphs, could offer a promising solution to this issue. In fact, efforts have been made to develop algorithms specifically designed for the analysis of plantar pressure parameters in distinct anatomical regions of the foot [38]. Furthermore, there have been reports of gait monitoring and fall detection systems incorporating automated algorithms for plantar pressure analysis, with some of these systems currently undergoing commercialization [39,40]. This progress in smart insoles has the potential to enable ongoing monitoring of the functional status and physical performance of the lower extremities of older individuals throughout their daily lives.

An important finding in this study is that the agreement between the two measurement methods was higher for the time measurements compared to the SPPB scores. Paradoxically, this emphasizes the critical importance of precise time measurements. Although the test–retest reliability of the SPPB-M was deemed acceptable, and its minimal detectable change was reported as 1.7 points, which is larger than the gap between the two measurement methods in this study [41], even slight variations in timing can result in corresponding increases in score discrepancies. This underscores the need for the development of a new, more accurate, and consistently repeatable method, instead of relying on a stopwatch prone to potential human error.

This study had some limitations. First, the SPPB was performed by a single examiner. Although we acknowledge the possibility of human error in this test, the examiner had over five years of experience conducting the SPPB according to standardized protocols, and no deviations from these protocols were made except for the use of a smart insole. To maintain accuracy, we conducted training sessions involving five participants and strictly adhered to standardized protocols during the assessment. Second, after converting the measured plantar pressure into a time–pressure graph, the interpreter noted the corresponding time based on the changes observed in the graph. To address potential concerns, two independent interpreters evaluated the graph to ensure high inter- and intra-interpreter reliability. Third, we failed to account for acceleration and deceleration during the gait speed test. Typically, the gait speed test designates approximately two feet at the start and end points as buffer zones, which are essential for capturing a stable and consistent gait speed. However, in our study, we neglected to include these buffer zones, relying exclusively on the data obtained from the FSR sensors, with the exception of the accelerometer sensor. The primary objective of this study was to assess variations in step patterns through changes in plantar pressure and to examine their potential applications in inspections. As indicated by the time–pressure graph, it is noteworthy that the sole analysis of changes in plantar pressure exhibited a strong correlation between the two measurement methods. Nevertheless, future research and the development of automated algorithms will necessitate the incorporation of acceleration sensor data. Finally, our study focused on 40 participants who, as evidenced by their basic characteristics, were relatively healthy. We conducted a normality distribution test, specifically the Shapiro–Wilk test, to assess the distribution of the total SPPB-SI and SPPB-M scores. The results confirmed that both scales followed a normal distribution pattern. However, this does not automatically imply the immediate application of smart insoles to all subjects. For the application of our research findings to patients of all ages and with various medical conditions, further studies targeting a larger and more diverse population are necessary.

## 5. Conclusions

The SPPB-SI, based on plantar pressure analysis using commercial smart insoles, demonstrated excellent agreement with the SPPB-M measured using a standardized protocol. These findings highlight the potential of smart insoles as reliable SPPB inspection devices. In the future, the use of automated algorithms to analyze plantar pressure graphs may further validate this possibility.

## Figures and Tables

**Table 1:** Characteristics of the study participants.

Characteristics	Total (N = 40)
Gender, women	24 (60%)
Age (years), mean (SD)	72.98 (9.27)
55–64	6 (15%)
65–74	11 (28%)
75–84	23 (57%)
BMI (kg/m^2^), mean (SD)	23.51 (2.33)
K-FRAIL score, mean (SD)	1.55 (1.13)
Grip strength (kg), mean (SD)	19.95 (8.89)
K-MBI score, mean (SD)	86.83 (16.74)
K-BBS score, mean (SD)	43.55 (12.61)
FAC score, mean (SD)	4.03 (1.23)
K-MMSE score, mean (SD)	27.15 (1.78)
SPPB-SI score, mean (SD)	
Total score	7.72 (2.50)
Balance test score	3.76 (0.63)
Gait speed test score	2.08 (1.19)
Five chair stand test score	2.13 (1.34)
SPPB-M score	
Total score	7.95 (2.63)
Balance test score	3.68 (0.73)
Gait speed test score	2.18 (1.17)
Five chair stand test score	2.25 (1.26)

Abbreviations: BMI, body mass index; FAC, functional ambulatory category; K-BBS, Korean version of the Berg Balance Scale; K-FRAIL, Korean version of the Fatigue, Resistance, Ambulation, Illness, and Loss of Weight scale; K-MBI, Korean version of the modified Barthel index; K-MMSE, Korean Mini-Mental State Examination; SD, standard deviation; SPPB-SI, Short Physical Performance Battery measured using smart insoles; SPPB-M, Short Physical Performance Battery manually measured using a stopwatch.

**Table 2:** Intra- and inter-interpreter reliability of the SPPB measured through plantar pressure graph interpretation using smart insoles.

	Intra-Interpreter Reliability	Inter-Interpreter Reliability
SPPB-SI times		
Balance test times	0.989 *	0.957 *
Gait speed test times	0.996 *	0.993 *
Five chair stand test times	0.999 *	0.999 *
SPPB-SI scores		
Balance test scores	0.889 *	0.889 *
Gait speed test scores	0.868 *	0.868 *
Five chair stand test scores	0.899 *	0.865 *

Note: score values were tested using the κ correlation coefficient, and time values were tested using the Pearson correlation coefficient. * *p* < 0.001. Abbreviations: SPPB, Short Physical Performance Battery; SPPB-SI, Short Physical Performance Battery measured using smart insoles.

**Table 3:** Correlation between SPPB scores (measured using smart insoles and a stopwatch) and geriatric functional parameters.

	K-FRAIL	K-BBS	FAC	K-MBI	Grip Strength	K-MMSE
SPPB-SI						
Total score	−0.333 (0.036)	0.686 (<0.001)	0.829 (<0.001)	0.743 (<0.001)	0.260 (<0.105)	0.303 (<0.058)
Balance test score	−0.272 (0.094)	0.360 (0.025)	0.516 (0.001)	0.432 (0.006)	0.246 (0.131)	0.091 (0.584)
Gait speed test score	−0.349 (0.027)	0.620 (<0.001)	0.775 (<0.001)	0.742 (<0.001)	0.276 (0.085)	0.194 (0.230)
Five chair stand test score	−0.165 (0.310)	0.658 (<0.001)	0.737 (<0.001)	0.632 (<0.001)	0.201 (0.215)	0.365 (0.021)
SPPB-M						
Total score	−0.355 (0.025)	0.678 (<0.001)	0.836 (<0.001)	0.737 (<0.001)	0.233 (0.148)	0.304 (0.056)
Balance test score	−0.342 (0.031)	0.424 (0.006)	0.554 (<0.001)	0.471 (0.002)	0.156 (0.338)	0.094 (0.565)
Gait speed test score	−0.419 (0.007)	0.682 (<0.001)	0.801 (<0.001)	0.749 (<0.001)	0.289 (0.071)	0.199 (0.219)
Five chair stand test score	−0.220 (0.172)	0.571 (<0.001)	0.697 (<0.001)	0.568 (<0.001)	0.157 (0.334)	0.401 (0.010)

Note: data are presented as ρ (*p*-value). ρ and *p*-value were obtained using the Spearman’s test. Abbreviations: FAC, functional ambulatory category; K-BBS, Korean version of the Berg Balance Scale; K-FRAIL, Korean version of the Fatigue, Resistance, Ambulation, Illness, and Loss of Weight scale; K-MBI, Korean version of the modified Barthel index; K-MMSE, Korean Mini-Mental State Examination; SPPB-SI, Short Physical Performance Battery measured using smart insoles; SPPB-M, Short Physical Performance Battery manually measured using a stopwatch.

## Data Availability

The data presented in this study are available on request from the corresponding author.
